# Non-motor symptoms questionnaire-based profiles in early-stage dementias: A prospective cohort study

**DOI:** 10.1177/13872877261487345

**Published:** 2026-09-21

**Authors:** Mika Kinnunen, Emma Vitikka, Johanna Krüger, Dorit Hoffmann, Annakaisa Haapasalo, Alberto Lleó, Olivia Belbin, Noora-Maria Suhonen, Barbara Borroni, Anne M. Portaankorva, Laura Säisänen, Kasper Katisko, Eino Solje

**Affiliations:** 1 Institute of Clinical Medicine – Neurology, 163043 University of Eastern Finland, Kuopio, Finland; 2 Research Unit of Clinical Medicine, Neurology, 6370 University of Oulu, Oulu, Finland; 3 577217 Medical Research Center, Oulu University Hospital, Oulu, Finland; 4 Neurocenter, Neurology, Oulu University Hospital, Oulu, Finland; 5 A.I Virtanen Institute for Molecular Sciences, 163043 University of Eastern Finland, Kuopio, Finland; 6 Sant Pau Memory Unit, IR SANT PAU 16689 Hospital de la Santa Creu i Sant Pau, Barcelona, Spain; 7 Centro de Investigación Biomédica en Red en Enfermedades Neurodegenerativas ( 145219 CIBERNED), Madrid, Spain; 8 Department of Clinical and Experimental Sciences, 9297 University of Brescia, Brescia, Italy; 9 Molecular Markers Laboratory, 18518 IRCCS Istituto Centro San Giovanni di Dio Fatebenefratelli, Brescia, Italy; 10 Clinical Neurosciences, 3835 University of Helsinki, Helsinki, Finland; 11 Neuro Center – Neurology, Kuopio University Hospital, Kuopio, Finland

**Keywords:** Alzheimer's disease, dementia with Lewy bodies, early diagnosis, frontotemporal dementia, non-motor symptoms

## Abstract

**Background:**

Current knowledge regarding the nature and prevalence of non-motor symptoms across different types of dementia is limited. While Alzheimer's disease (AD), behavioral variant frontotemporal dementia (bvFTD), and dementia with Lewy bodies (DLB) are primarily characterized by cognitive and motor symptoms, several other features such as non-motor symptoms are also known to occur in all these conditions.

**Objective:**

To assess non-motor symptoms in early-stage dementia and characterize symptom profiles across different neurodegenerative dementia diseases.

**Methods:**

A total of 64 AD, 38 bvFTD, and 17 DLB early-stage patients, along 63 cognitively healthy controls (HC), were prospectively recruited from Kuopio and Oulu University Hospitals. Non-motor symptoms were evaluated using the Non-Motor Symptoms Questionnaire (NMSQ), focusing on gastrointestinal, neuropsychiatric, cognitive, sleep-related and sensory-motor domains within three timeframes prior to the baseline visit.

**Results:**

DLB patients exhibited the highest prevalence of non-motor symptoms across all domains. bvFTD and AD patients did not show disease group specific symptoms, but apathy and paranoidal thoughts appeared earlier in bvFTD than AD or DLB. Diplopia was reported by 14% of DLB patients (Fisher exact test, *p* = 0.035) one year before diagnosis. Neuropsychiatric and cognitive symptoms were more frequent in all dementia groups compared to HC (Fisher exact test, *p* < 0.01).

**Conclusions:**

Non-motor symptom profiles differ among these dementia subtypes even at early stages, suggesting their potential utility as early disease-specific indicators.

## Introduction

Neurodegenerative dementias, including frontotemporal dementia (FTD), Alzheimer's disease (AD), and dementia with Lewy bodies (DLB), pose a major global challenge to healthcare systems due to diagnostic complexity, limited treatment options and the substantial burden these conditions place on patients and caregivers. These three disease groups represent the most common forms of neurodegenerative dementia, each with distinct clinical, neuropathological, and neurochemical profiles.^1–3^ Pathophysiologically, AD is primarily associated with cholinergic dysfunction and the accumulation of amyloid-β and tau in the brain, with downstream cholinergic deficits.^4,5^ In contrast, FTD is typically characterized by TDP-43 or tau brain aggregates and is linked to disruptions of serotonergic, GABAergic and glutamatergic neurotransmitter systems, with a relatively well-preserved cholinergic function.^6–8^ DLB is characterized by alpha-synuclein brain deposition and widespread neurotransmitter deficits, including cholinergic, glutamatergic and GABAergic dysfunction.^9,10^ Agreeing with the observed neurotransmitter deficits in the different dementias, our recent study indicated alterations in the levels of specific synaptic proteins (VAMP-2 and SNAP-25), which affect neurotransmitter release at the synapses, in the cerebrospinal fluid of pure DLB patients and DLB patients with concomitant AD neuropathology.^11^ These alterations were also associated with cognitive changes in these patient groups, further underscoring the contribution of neurotransmitter dysfunction in disease pathogenesis.^11^ Altogether, these neurophysiological and biological differences might explain the clinical heterogeneity among these dementing disorders.

Early and accurate diagnosis of AD, FTD, and DLB remains challenging especially due to overlapping cognitive symptoms and limitations in biomarker specificity.^12^ Furthermore, neuropsychiatric symptoms such as depression, anxiety, apathy, and psychotic symptoms are common across dementia types and often lead to misdiagnoses.^13–17^

While cognitive and motor symptoms are well described in these conditions, other features such as non-motor symptoms remains less well understood. In DLB, symptoms such as orthostatic hypotension, constipation, olfactory deficits, and erectile dysfunction are relatively well documented.^3,18,19^ In AD, impaired vagal activity and altered cardiovascular regulation have been noted,^20^ whereas in FTD, symptoms such as temperature dysregulation, altered sweating, urinary incontinence and flushing, have been reported.^21^ However, prior studies have not systematically compared the wide range of potential non-motor symptoms across these three dementia subtypes.

We hypothesized that already in early disease stages, AD, bvFTD, and DLB present with distinct non-motor symptom profiles. Identifying these differences may aid the differential diagnostics and improve our understanding of disease mechanisms. Therefore, the aim in this study was to systematically characterize these symptoms using a Non-motor Symptoms Questionary survey (NMSQ), originally developed for Parkinson's disease,^22^ in a prospectively recruited cohort of patients with early-stage dementia patients and healthy controls.

## Methods

### Study participants

We prospectively recruited all referred patients with a suspected early neurodegenerative disorder, who provided a written informed consent at the memory clinics of Kuopio University Hospital (KUH) and Oulu University Hospital (OUH) between 2022–2025. The study was approved by the regional ethics committee of the Northern Savonia Hospital District (2130/2021). Patients that were included in this study received an at least probable diagnosis of AD, bvFTD, or DLB, supported by brain imaging data according to the most recent criteria used in Finland do diagnose these neurodegenerative diseases.^23–25^ Early-stage neurogenerative disease was defined as a Clinical Dementia Rating (CDR)^26^ global score of 0–2 at the first specialist visit. CDR scores were retrospectively collected from medical records and were used to characterize disease stage rather than to establish the diagnosis itself. A small number of participants had CDR score of 2 at the first specialist visit. These individuals were included because the study aimed to capture patients presenting to memory clinics near the time of diagnosis and the first assessment rather than to apply a strict CDR-based disease-stage cutoff. Total number of dementia patients included in this study was 119 and the distribution between separate disease groups were as follows: 64 AD, 38 bvFTD, and 17 DLB patients. We excluded motor manifestations of FTD (FTD-ALS, Progressive supranuclear palsy, and corticobasal syndrome patients) due to very small sample sizes. Participants completed the NMSQ as part of this scientific

study, and the results were not used in the diagnostic process. Patients were examined according to the standard clinical protocol used, including clinical examination, comprehensive neuropsychological examination, and brain MRI and/or FDG-PET scan. Cerebrospinal fluid (CSF) AD biomarkers were inspected if there was no contraindication for a lumbar puncture. Of the AD patients, 97% had biomarker (CSF or amyloid-PET) based diagnose. AD-type CSF pathology led to exclusion from the bvFTD and DLB groups in this study. In addition to the neurodegenerative disease groups, healthy controls (HC, N = 63) were recruited as a control group. Participants in the HC group were confirmed to be cognitively healthy by normal Consortium to Establish a Registry for Alzheimer's Disease neuropsychological battery (CERAD-NB) test results,^27^ they had no subjective memory complaints and were capable of normal daily functions.

Comorbidities of the whole cohort were collected retrospectively from medical records near at the NMSQ evaluation. Comorbidities refer to conditions which the participants have had prior to taking part in the study. Of the comorbidities, ten different types were screened; 1) Cardiovascular (hypertension, coronary artery disease, arteriosclerosis etc.); 2) Metabolic and Endocrinological (type I or II diabetes, hypercholesterolemia, hypothyroidism or hyperthyroidism, gout etc.); 3) Previously cured any type of cancer; 4) Other neurologic (epilepsy, migraine, multiple sclerosis, previous transient ischemic attack, small clinically asymptomatic ischemic infarct on imaging etc.); 5) Respiratory system related (asthma, COPD, sleep apnea etc.); 6) Sleep related (restless leg syndrome, non-organic insomnia etc.); 7) GI-tract related (IBD, reflux disease, celiac disease etc.); 8) Musculoskeletal related (any osteoarthritis, osteoporosis etc.); 9) Past psychiatric disorder (ADHD, depression, schizophrenia, etc.), and 10) Eye related (cataract).

### Symptom assessment

The NMSQ is a self-completed, dichotomous yes/no questionnaire initially designed to screen for non-motor symptoms in Parkinson's disease.^22^ Patients and HC filled the NMSQ with 30 questions in total, early symptoms experienced during the preceding two-year before the baseline visit were characterized. We grouped these 30 items into four categories, aligned with the higher-level domains assessed by the NMSQ. The categories comprised gastrointestinal symptoms, encompassing gastrointestinal and urinary function; neuropsychiatric and cognitive symptom, covering aspects related to memory, cognition and mood; sleep-related symptoms; and other sensory-motor symptoms, which included heterogeneous set of manifestations such as pain, falls, diplopia and questions related to sexual functioning. If needed, the patients were allowed to receive assistance from their spouses or other close ones when completing the questionnaire, for example sleep related symptoms. In the Finnish version of the NMSQ, respondents were instructed to indicate whether each symptom had occurred during the past 3 months, past year or past two years. Unanswered question resulted in the exclusion of the patient just from that particular analysis.

For analysis, NMSQ items were grouped into four composite symptom categories based on symptom type: 1) gastrointestinal system symptoms (items 1–9 + 11), 2) neuropsychiatric and cognitive symptoms (items 12–17 + 30), 3) sleep-related symptoms (items 22–26) and 4) other sensory-motor symptoms (items 10, 18–21 and 27–29). Further analyses were also then performed considering each question separately.

### Statistical analyses

Symptoms present based on the NMSQ were compared between each disease group and HC and were analyzed with the Chi-Square test (*χ*^2^-test) or Fisher's exact test when appropriate, i.e., if the expected frequency was below one and below 20% of expected frequencies was smaller than five. Also, Phi Coefficient was used to evaluate association between two dichotomous variables. In this comparison, values close to 1 indicates very strong relationship, and values close to 0 are indicating weak relationship. To evaluate the robustness of the observed significant differences in the initial analyses, binary logistic regression analysis was further used to adjust for differences in sex and age distributions between the groups. The regression analysis was used when possible or appropriate, noting that small sample sizes in the groups affected their reliability (mostly in the DLB group comparisons). p-values less than 0.05 were considered statistically significant. Statistical analyses were performed with IBM SPSS software version 29.

## Results

### Prevalence of NMSQ symptoms in different disease groups

Of the whole cohort, 105 were women (58%). Of the participants 104 (57%) were from KUH and 78 (43%) participants were from OUH. Differences in age, sex, CDR-score and comorbidities across the studied dementia groups was evaluated and presented. Table 1 shows the distribution between participants among different dementia groups and controls.

**Table 1.** Basic characteristics of the study cohort at NMSQ evaluation.

	AD N = 64	bvFTD N = 38	DLB N = 17	HC N = 63
Age, mean, (SD) [Range]	64.1^b*^ (6.3) [44-79]	62.5 (5.2) [51-72]	71.2^c**^ (6.7) [54-82]	59.0 (11.5) [32-77]
Sex (males%)	50%^b*^	45%^a*^	82%^c**^	22%
CDR Score, mean, (SD) [Range]	0.77 (0.41) [0-2]	0.88 (0.42) [0.5-2]	0.71 (0.47) [0-2]	NA
Comorbidities				
Cardiovascular	56%	63%	70%	34%
Metabolic and Endocrinological	42%	47%	41%	25%
Cancer	5%	3%	0%	10%
Other neurologic	23%^b*^	32%^a*^	0%	5%
Respiratory system	20%	18%	6%^c*^	25%
Sleep related	3%	3%	6%	0%
GI-tract related	3%	3%	12%	5%
Musculo-skeletal	17%	13%	12%	14%
Psychiatric	5%	13%	0%	3%
Eye-related	0%	5%	12%^c*^	0%

The effect of age and comorbidities was tested with *χ*^2^ and sex and CDR-score using Mann-Whitney U test. ^a^bvFTD versus HC, ^b^AD versus HC, ^c^DLB versus HC. Only statistically significant *p*-values (*p* < 0.05) between specific groups are indicated. *p < 0.05, **p < 0.001.

CDR scores were obtained retrospectively from the first memory clinic visit and are presented to describe disease severity. Diagnostic classification was based on established clinical criteria and supportive investigations rather than CDR scores. Patients with recorded CDR score of 0 fulfilled diagnostic criteria for neurodegenerative disease.

AD: Alzheimer’s disease; bvFTD: behavioral variant frontotemporal dementia; DLB: dementia with Lewy bodies; HC: healthy control; NA: not available, NMSQ: Non-motor Symptoms Questionnaire survey; SD: standard deviation.

First, we evaluated NMSQ symptoms grouped into four composite categories in the NMSQ: gastrointestinal system symptoms (items 1–9 and 11), neuropsychiatric and cognitive symptoms (items 12–17, and 30), sleep-related symptoms (items 22–26), and other sensory-motor symptoms (items 10, 18–21 and 27–29) (Table 2), and after we examined each NMSQ item individually across all time points (Supplemental Tables 1–4). Overall, DLB patients had the highest prevalence of symptoms across all categories, followed by bvFTD and AD patients. Regarding the comparison of neuropsychiatric and cognitive symptoms, age (but not sex) was a significant confounding factor in the comparisons between bvFTD and HC, as well as AD and HC. Neither sex nor age had a statistically significant effect on the comparison other sensory motor symptoms between AD and HC.

**Table 2.** Prevalence of NMSQ symptom categories in AD, bvFTD, and DLB patients and HC

Symptom category	AD N = 64	bvFTD N = 38	DLB N = 17	HC N = 63	*χ*^2^ or Fischer’s exact test *p*	Binary logistic regression OR, *p* [95% Cl]
Gastrointestinal system	66%	67%	100%	75%	<0.05^a,b^ 0.018^c^	NA^c^
Neuropsychiatric & Cognitive	79%	82%	100%	51%	<0.01^a,b^ <0.001^c^	5.13, 0.001, [1.88–14.0]^a^ 2.45, <0.001, [1.55–3.87]^b^ NA^c^
Sleep-related	44%	46%	88%	65%	>0.05^a,c^ 0.020^b^	0.69, 0.057, [0.46–1.01]^b^ 1.86, 0.043, [1.02–3.40]^c^
Other sensory motor	58%	72%	100%	73%	>0.05^a,b^ 0.017^c^	0.63, 0.034, [0.41–0.97]^b^ NA^c^

The data were analyzed with *χ*^2^ or Fischer’s exact test and binary logistic regression. Odds ratio (OR) and 95% confidence intervals (CI) are reported. ^a^bvFTD versus HC, ^b^AD versus HC, ^c^DLB versus HC. The OR with 95% CI and *p-*values are shown in the separate columns of each disease group and represent logistic regression analysis between the disease group and HC adjusted for age and sex. AD: Alzheimer’s disease; bvFTD: behavioral variant frontotemporal dementia; DLB: dementia with Lewy bodies; HC: healthy control; NA: not available.

### Gastrointestinal system symptoms

DLB patients consistently reported the highest prevalence of gastrointestinal system-related symptoms, including daytime saliva dribbling, olfactory dysfunction, constipation, and unexpected weight changes to the other groups. Constipation (50% versus 15%, *p* = 0.006, Phi Coefficient 0.34), and olfactory dysfunction (38% versus 7%, *p* = 0.007, Phi Coefficient 0.38) occurred more often already two years before the DLB diagnosis as compared to the HC group.

In the DLB group, daytime saliva dribbling (18% versus 0%, *p* = 0.009, Phi Coefficient 0.38) and unexpected weight changes (27% versus 3%, *p* = 0.014, Phi Coefficient 0.34) were more common compared to HC group and occurred one year before DLB diagnosis (18% versus 0%, *p* = 0.009, Phi Coefficient 0.38).

No statistically significant differences were observed between DLB and HC for dysphagia, nausea, fecal incontinence, incomplete bowel emptying and urinary problem, and there were none statistically significant differences when AD and bvFTD were compared to HC. Full data on the prevalence of gastrointestinal system symptoms in each group are presented in Supplemental Table 1.

### Neuropsychiatric and cognitive symptoms

The DLB group reported most often neuropsychiatric and cognitive symptoms in comparison to the other disease groups or HC. Memory complaints were reported in every disease group already two years before the diagnosis: 42% in bvFTD (*p* = 0.002, Phi Coefficient 0.35), 45% in AD (*p* < 0.001, Phi Coefficient 0.37), and 39% in DLB patients (*p* = 0.034, Phi Coefficient 0.28) compared to 12% reported by the HC.

Loss of interest in the surroundings was more prevalent in bvFTD 2 years before diagnosis (41% versus 10%, *p* = 0.001, Phi Coefficient 0.37) when compared to HC. In AD (29%, *p* = 0.033, Phi Coefficient 0.21) and DLB (56%, *p* < 0.001, Phi Coefficient 0.44) groups it occurred one year before diagnosis compared to 12% of HC.

Audiovisual hallucinations were experienced in every disease group, but in different timeframes. In the DLB, 2 years before diagnosis (20%, *p* = 0.007, Phi Coefficient 0.41), in the bvFTD, 1 year before diagnosis (14%, *p* = 0.008, Phi Coefficient 0.38) and in the AD group, three months before diagnosis (9%, *p* = 0.024, Phi Coefficient 0.22), whereas it was absent in HC group in every timepoint.

Concentration problems occurred in AD (48%, *p* = 0.010, Phi Coefficient 0.26) and bvFTD group (62%, *p* < 0.001, Phi Coefficient 0.38) one year before diagnosis compared to 22% reported by the HC. In the DLB group concentration problems occurred 3 months before diagnosis (67% versus 26%, *p* = 0.005, Phi Coefficient 0.40) when compared to HC.

Paranoidal thoughts were also reported in every disease group, but significant differences were observed in different timepoints. In bvFTD it occurred already two years before diagnosis (9%, *p* = 0.047, Phi Coefficient 0.25) when compared to HC. In AD (11%, *p* = 0.010, Phi Coefficient 0.25), and DLB group (33%, *p* < 0.001, Phi Coefficient 0.54) it occurred one year before diagnosis compared to HC, where it was absent in every timepoint. Depression was more prevalent in DLB group one year before diagnosis (63% versus 28%, *p* = 0.020, Phi Coefficient 0.28) when compared to HC.

Full data on the prevalence of neuropsychiatric and cognitive symptoms in each group are presented in Supplemental Table 2.

### Sleep-related symptoms

Patients in the DLB group reported most often sleep-related symptoms in comparison to the other disease groups or HC. In the DLB group, especially vivid dreams (54% versus 18% *p* = 0.013, Phi Coefficient 0.32) and daytime fatigue (20% versus 2% *p* = 0.025, Phi Coefficient 0.33) were more prevalent one year before diagnosis and restless sleep (31% versus 5% *p* = 0.016, Phi Coefficient 0.34) was more prevalent already two years before the diagnosis compared to HC. In the AD group, patients had less problems to fall asleep than HC in every time point*.* Between bvFTD and HC there were no statistically significant differences in any comparison. Full data on the prevalence of sleep-related symptoms in each group are presented in Supplemental Table 3.

### Other sensory-motor symptoms

The DLB patients reported most often other sensory-motor symptoms overall when compared to the other disease groups. Difficulties to have sex when trying was more prevalent in the DLB group two years before diagnosis (57% versus 13%, *p* = 0.001, Phi Coefficient 0.44). Feeling lightheaded or dizzy after standing up was also only more prevalent in DLB group (42% versus 13%, *p* = 0.030, Phi Coefficient 0.28), and it was reported three months before the diagnosis.

Fallings were more prevalent in bvFTD (24%, *p* = 0.009, Phi Coefficient 0.29) and DLB (27%, *p* = 0.025, Phi Coefficient 0.30) groups two years before the diagnosis, when compared to 5% reported by HC.

In the AD group there was less reported interest in sex, leg swelling and sweating than HC group. The lack of prevalence of leg swelling was less common one year before the diagnosis, whereas the lack of sweating and interest in sex occurred two years before the diagnosis.

Double visions were reported in AD (7%, *p* = 0.049, Phi Coefficient 0.20) and DLB group (14%, *p* = 0.035, Phi Coefficient 0.25) one year before the diagnosis, but in the DLB group significant difference was also observed three months before diagnosis (25%, *p* = 0.004, Phi Coefficient 0.47), whereas it was absent in HC group in every timepoint. No statistically significant differences were observed in unexplained pain in any disease group versus HC.

Full data on the prevalence of other sensory-motor symptoms in each group are presented in Supplemental Table 4.

## Discussion

In this study, we systematically evaluated diverse range of early non-motor symptoms in AD, bvFTD, and DLB patients and HC using NMSQ. To our knowledge, this is the first study to apply a standardized instrument like the NMSQ across all of these three major neurodegenerative dementia subtypes in a prospective cohort. Our findings reveal distinct symptom patterns between the dementia groups, particularly a greater burden of non-motor symptoms in the DLB patients, and several group-specific features regarding symptom presentation or time frames, that may offer diagnostic value and deepen our understanding of the disease pathophysiological processes. Importantly, many of the symptoms captured by the NMSQ reflect non-specific manifestations that may accompany parkinsonism and other neurodegenerative disorders, rather than Lewy body pathology alone. Because parkinsonian features are not unique to Lewy body spectrum disorders, similar non-motor symptoms may also occur in other dementias. Therefore, the presence of these symptoms in isolation should not be interpreted as evidence of a Lewy body disorder. Instead, their diagnostic value should be evaluated alongside the overall clinical presentation and other disease-specific characteristics. Recognizing this overlap is essential to avoid misleading diagnostic pathways and to support accurate differential diagnosis already in the early stages of disease.

The prominent symptom presentation (including gastrointestinal, sleep-related and other sensory-motor symptoms) in DLB patients is consistent with previous research. In the present study, DLB patients frequently reported olfactory dysfunction, constipation, and lightheaded feeling after standing up. These findings are well in concordance with earlier studies documenting gastrointestinal, olfactory loss and cardiovascular autonomic dysfunction in DLB patients.^18,19,28^ We also found that 27% of the DLB patients experienced falls and 20% audiovisual hallucinations two years before diagnosis. Prior literature suggests that visual hallucinations are a risk factor for falls in DLB.^29^ Notably, 14% of the DLB patients had diplopia for one year prior and 25% reported it three months prior to the diagnosis, an observation not previously described. It is also noticeable, that prevalence of cataract was significantly higher in DLB group than HC group in this study, but only 12%, which is 2 DLB patients, reported it while prevalence of diplopia was 25% in one year prior. Also, both patients with reported cataract, did not report diplopia in the questionary. While diplopia is not a previously recognized feature of prodromal DLB, our findings suggest that it may warrant further investigation as a potential early marker of the disease.

In bvFTD and AD patients, we did not find any group specific non-motor symptom. In the bvFTD symptoms like loss of interest in surroundings, and paranoidal thoughts appeared earlier than AD or DLB. These findings are suggested by one earlier study, which found delusions and hallucinations as a prodromal symptom of the bvFTD.^16^ One of the core symptoms of bvFTD is apathy, which is also listed as one of the diagnostic criterias.^23^ Our findings are strengthening evidence of apathy to be prodromal symptom of bvFTD.

Contrary to previous findings, we observed less frequent self-reported sleep problems in AD patients than in HC. Only about one fifth of AD patients reported difficulties in falling or staying asleep, compared to nearly half of the HC group. This contrasts with studies reporting disturbed sleep continuity in AD.^30^ The divergence may be due to methodological differences since our study relied on self-reported symptoms rather than polysomnographic or objective sleep assessments.

Analyses revealed no statistically significant differences between the groups across multiple symptom measures. For example, symptoms, such as dysphagia, hyperhidrosis, and urinary incontinence, were not significantly more prevalent in any specific group, although these have been reported in bvFTD, and DLB patients in other studies.^21,31^ This discrepancy may relate to the differences in disease stage. In the study by Ahmed et al., patients had a mean disease duration of 6.4 years,^21^ whereas our sample consisted of early-stage cases. Additionally, we excluded patients with motor subtypes of the FTD spectrum, in whom dysphagia is more common.^32^ It is noticeable, that AD patients reported less leg swelling and less excessive sweating than controls. By previous study, it is unknown whether thermoregulation dysfunction appears before or after known symptoms of AD.^33^ Our findings suggest that these symptoms should not be considered early non-motor symptoms of AD, although further investigation is needed.

We did not observe an increased prevalence of symptoms including fecal incontinence, unexplained pain, urinary incontinence, and leg swelling, in any of the dementia groups compared with HC. Previous literature has reported somatic complaints in FTD and high prevalence of pain in AD.^34,35^ However, pain is often underreported in self-assessments, especially in patients with cognitive impairment, and may be better captured through proxy reporting or observational tools.^36^

It is noteworthy that for certain symptoms, such as diplopia in patients with AD and daytime fatigue in patients with DLB, the observed prevalence was higher one year prior to diagnosis, but not two years or three months before diagnosis. This pattern may be explained, for example by fluctuations in patients’ symptomatology during the two-year period preceding the diagnosis. On the other hand, this may be explained by self-reporting bias, although caregiver assistance was allowed. It is also important to notice that propensity score matching was not done due to the limited cohort size, which may have induced some results.

The relatively high prevalence of symptom endorsement across all domains in the HC group warrants careful interpretation. This finding likely reflects the design of the NMSQ as a high-sensitivity screening instrument, capturing a broad range of common and often non-specific symptoms that are prevalent in general aging population. Consequently, the presence of at least one symptom within a domain does not necessarily indicate underlying pathology but rather limits the specificity of domain level prevalence estimates. In this context, the interpretative value of questionnaire lies not in domain-level classification but in the prevalence of individual symptoms.

This study has several strengths, including a prospective design, use of a standardized questionnaire with modification only regarding lengthening the studied time period, which gave opportunity to evaluate symptoms from longer period before diagnosis, and inclusion of three major dementia subtypes diagnosed according to current criteria,^23–25^ and cognitively healthy controls. Also, wide range of comorbidities were screened, which may influence non-motor and autonomic symptoms, and signific differences were observed in other neurologic diseases and cataract prevalences. However, the limitations include the small size of the DLB group, which reduced statistical power and limited the robustness of some comparisons. There were also age and sex differences between the groups, although these were adjusted for in the regression models, but due to small sample size, statistical power was reduced, and due to the exploratory nature of the study, statistical corrections for multiple comparisons were not used, acknowledging the risk of type 1 error. Additionally, we did not include any physiological measures, no medication use was evaluated in this study, and NMSQ scale is only validated for Parkinson's disease, so sensitivity and specificity may variate between different disease groups. Finally, self-reporting is prone to recall bias, even though the option for caregiver assistance, if needed, in filling in the questionnaire likely reduced this risk. However, such symptoms are typically assessed using patient-reported questionnaires at the stage when diagnostic evaluations are initiated. Therefore, it is important to understand how these instruments perform in disease groups other than primary Lewy body spectrum disorders.

### Conclusion

This study demonstrates that early-stage AD, bvFTD, and DLB are associated with distinct non-motor symptom profiles. DLB shows the highest symptom burden across all domains, while bvFTD is showing apathy and paranoidal thoughts earlier than other dementia groups. These findings highlight the potential diagnostic value of systematic non-motor symptom assessment in different dementias and suggest that these features may reflect underlying pathophysiological differences in the disease processes. In addition, they may improve our understanding of the clinical manifestations across the disease spectrum during the early and prediagnostic stages. Future longitudinal studies with larger sample size, age-matched patient groups, and in addition to physiological measurements of the symptoms are needed to confirm and expand the findings of this study.

## Supplemental Material

sj-docx-1-alz-10.1177_13872877261487345 - Supplemental material for Non-motor symptoms questionnaire-based profiles in early-stage dementias: A prospective cohort study Supplemental material, sj-docx-1-alz-10.1177_13872877261487345 for Non-motor symptoms questionnaire-based profiles in early-stage dementias: A prospective cohort study by Mika Kinnunen, Emma Vitikka, Johanna Krüger, Dorit Hoffmann, Annakaisa Haapasalo, Alberto Lleó, Olivia Belbin, Noora-Maria Suhonen, Barbara Borroni, Anne M. Portaankorva, Laura Säisänen, Kasper Katisko and Eino Solje in Journal of Alzheimer's Disease

## Data Availability

The data supporting the findings of this study are available on reasonable request from the corresponding author. The data are not publicly available due to privacy or ethical restrictions.
