# Supplementary material for: Non-motor symptoms questionnaire-based profiles in early-stage dementias: A prospective cohort study
Source: J Alzheimers Dis. 2026 Sep 21;114(1):267–75. doi: 10.1177/13872877261487345 (PMC13675529; doi:10.1177/13872877261487345)
Supplement: Supplementary file 1 [file sj-docx-1-alz-10.1177_13872877261487345.docx]

**Supplemental Material**

**Non-motor symptoms questionnaire-based profiles in early-stage dementias: A prospective cohort study**

**Supplemental Table 1.** Gastrointestinal system symptoms experienced during the past 3 months, past year and past two years in the different disease groups at different times before the diagnosis versus healthy controls at different times before filling in the questionnaire.

|  | AD  N = 64 | bvFTD  N = 38 | DLB  N = 17 | HC  N = 63 |
| --- | --- | --- | --- | --- |
| 1. Dribbling of saliva during daytime (%) | | | | |
| 3 months | 2 | 3 | 0 | 0 |
| 1 year | 0 | 5 | 18 ^(^*^p^* ^= 0.009)^ | 0 |
| 2 years | 2 | 8 | 7 | 0 |
| 2. Loss or change in the ability to taste or smell (%) | | | | |
| 3 months | 5 | 14 | 38 ^(^*^p^* ^< 0.001)^ | 3 |
| 1 year | 5 | 11 | 41 ^(^*^p <^* ^0.001^*^)^* | 5 |
| 2 years | 9 | 11 | 38 ^(^*^p^* ^= 0.005)^ | 7 |
| 3. Difficulty in swallowing food or drink or problems with choking (%) | | | | |
| 3 months | 3 | 5 | 6 | 6 |
| 1 year | 4 | 9 | 6 | 7 |
| 2 years | 3 | 8 | 0 | 9 |
| 4. Vomiting or feelings of sickness (nausea) (%) | | | |  |
| 3 months | 2 | 11 | 0 | 3 |
| 1 year | 7 | 14 | 0 | 3 |
| 2 years | 7 | 11 | 6 | 5 |
| 5. Constipation (less than 3 bowel movements a week) or having to strain to pass a stool (feces) (%) | | | | |
| 3 months | 12 | 19 | 57 ^(^*^p <^* ^0.001)^ | 10 |
| 1 year | 16 | 22 | 53 ^(^*^p =^* ^0.001^*^)^* | 12 |
| 2 years | 13 | 22 | 50 ^(^*^p =^* ^0.006)^ | 15 |
| 6. Bowel (fecal) incontinence (%) | | | | |
| 3 months | 3 | 11 | 13 | 7 |
| 1 year | 4 | 8 | 7 | 7 |
| 2 years | 0 | 11 | 7 | 3 |
| 7. Feeling that your bowel emptying is incomplete after having been to the toilet (%) | | | | |
| 3 months | 10 | 8 | 40 | 15 |
| 1 year | 4 | 8 | 36 | 14 |
| 2 years | 6 | 11 | 20 | 12 |
| 8. A sense of urgency to pass urine makes you rush to the toilet (%) | | | | |
| 3 months | 28 | 36 | 60 | 32 |
| 1 year | 20 | 37 | 50 | 35 |
| 2 years | 15 | 32 | 50 | 25 |
| 9. Getting up regularly at night to pass urine (%) | | | | |
| 3 months | 32 | 32 | 54 | 32 |
| 1 year | 30 | 25 | 60 | 31 |
| 2 years | 30 | 26 | 50 | 23 |
| 11. Unexpected change in weight (not due to change in diet) (%) | | | | |
| 3 months | 2 | 11 | 29 ^(^*^p^* ^= 0.011)^ | 3 |
| 1 year | 6 | 14 | 27 ^(^*^p^* ^= 0.014)^ | 3 |
| 2 years | 11 | 11 | 19 | 3 |

*p*-values from *χ*^2^ or Fischer’s exact test between specific disease group versus HC are shown.

AD: Alzheimer’s disease; bvFTD: behavioral variant frontotemporal dementia; DLB: dementia with Lewy bodies; HC: healthy control

**Supplemental Table 2.** Neuropsychiatric and cognitive symptoms experienced during the past 3 months, past year and past two years in the different disease groups at different times before the diagnosis versus healthy controls at different times before filling in the questionnaire.

|  | AD  N = 64 | bvFTD  N = 38 | DLB  N = 17 | HC  N = 63 | |
| --- | --- | --- | --- | --- | --- |
| 12. Problems in remembering things that have happened recently or  forgetting to do things (%) | | | | |  |
| 3 months | 71 ^(^*^p^* ^< 0.001)^ | 58 ^(^*^p <^* ^0.001)^ | 85 ^(^*^p <^* ^0.001)^ | 19 | |
| 1 year | 74 ^(^*^p^* ^< 0.001)^ | 53 ^(^*^p^* ^< 0.001)^ | 75 ^(^*^p^* ^< 0.001)^ | 16 | |
| 2 years | 45 ^(^*^p^* ^< 0.001)^ | 42 ^(^*^p^* ^= 0.002)^ | 39 ^(^*^p^* ^= 0.034)^ | 12 | |
| 13. Loss of interest in what is happening around you or doing things (%) | | | | |  |
| 3 months | 29 | 47 ^(^*^p^* ^= 0.001)^ | 43 ^(^*^p =^* ^0.028)^ | 15 | |
| 1 year | 29 ^(^*^p^* ^= 0.033)^ | 50 ^(^*^p^* ^< 0.001)^ | 56 ^(p < 0.001)^ | 12 | |
| 2 years | 8 | 41 ^(^*^p =^* ^0.001)^ | 14 | 10 | |
| 14. Seeing or hearing things that you know or are told not to be there (%) | | | | |  |
| 3 months | 9 ^(^*^p^* ^= 0.024)^ | 14 ^(^*^p^* ^= 0.006)^ | 31 ^(^*^p <^* ^0.001)^ | 0 | |
| 1 year | 4 | 14 ^(^*^p^* ^= 0.008)^ | 36 ^(^*^p <^* ^0.001)^ | 0 | |
| 2 years | 4 | 8 | 20 ^(^*^p^* ^= 0.007)^ | 0 | |
| 15. Difficulty in concentrating or staying focused (%) | | | | |  |
| 3 months | 45 ^(^*^p^* ^= 0.049)^ | 56 ^(^*^p^* ^= 0.005)^ | 67 ^(^*^p^* ^= 0.005)^ | 26 | |
| 1 year | 48 ^(^*^p^* ^= 0.010)^ | 62 ^(^*^p^* ^< 0.001)^ | 50 | 22 | |
| 2 years | 21 | 38 | 25 | 22 | |
| 16. Feeling sad, ‘’low’’, or ‘’blue’’ (%) | | | | |  |
| 3 months | 46 | 47 | 62 | 32 | |
| 1 year | 39 | 46 | 63 ^(^*^p^* ^= 0.020)^ | 29 | |
| 2 years | 24 | 37 | 17 | 22 | |
| 17. Feeling anxious, frightened, or panicky (%) | | | | |  |
| 3 months | 19 | 31 | 33 | 15 | |
| 1 year | 15 | 26 | 21 | 15 | |
| 2 years | 12 | 17 | 7 | 8 | |
| 30. Believing things are happening to you that other people say are not true (%) | | | | |  |
| 3 months | 12 ^(^*^p^* ^= 0.002)^ | 17 ^(^*^p^* ^< 0.001)^ | 23 ^(^*^p^* ^= 0.004)^ | 0 | |
| 1 year | 11 ^(^*^p^* ^= 0.010)^ | 12 ^(^*^p^* ^= 0.014)^ | 33 ^(^*^p^* ^< 0.001)^ | 0 | |
| 2 years | 4 | 9 ^(^*^p^* ^= 0.047)^ | 7 | 0 | |

*p*-values from *χ*^2^ or Fischer’s exact test between specific disease group versus HC are shown.

AD: Alzheimer’s disease; bvFTD: behavioral variant frontotemporal dementia; DLB: dementia with Lewy bodies; HC: healthy control

**Supplemental Table 3.** Sleep-related symptoms experienced during the past 3 months, past year and past two years in different disease groups at different times before the diagnosis versus healthy controls at different times before filling in the questionnaire.

|  | AD  N = 64 | bvFTD  N = 38 | DLB  N = 17 | HC  N = 63 |
| --- | --- | --- | --- | --- |
| 22. Finding it difficult to stay awake during activities, such as working, driving, or eating (%) | | | | |
| 3 months | 2 | 3 | 8 | 2 |
| 1 year | 0 | 0 | 20 ^(^*^p^* ^= 0.025)^ | 2 |
| 2 years | 2 | 0 | 7 | 2 |
| 23. Difficulty in falling to sleep or staying asleep at night (%) | | | | |
| 3 months | 18 ^(^*^p^* ^= 0.001)^ | 36 | 31 | 48 |
| 1 year | 16 ^(^*^p^* ^< 0.001)^ | 35 | 39 | 49 |
| 2 years | 15 ^(^*^p^* ^< 0.001)^ | 31 | 21 | 46 |
| 24. Intense, vivid, or frightening dreams (%) | | | | |
| 3 months | 9 | 8 | 64 ^(^*^p^* ^< 0.001)^ | 15 |
| 1 year | 6 | 8 | 54 ^(^*^p^* ^= 0.013)^ | 18 |
| 2 years | 6 | 8 | 36 | 17 |
| 25. Talking or moving about in your sleep as if you were ‘’acting’’ out a dream (%) | | | | |
| 3 months | 7 | 14 | 54 ^(^*^p^* ^< 0.001)^ | 3 |
| 1 year | 9 | 14 | 62 ^(^*^p^* ^< 0.001)^ | 5 |
| 2 years | 9 | 8 | 31 ^(^*^p^* ^= 0.016)^ | 5 |
| 26. Unpleasant sensations in your legs at night or while resting and feeling that you need to move (%) | | | | |
| 3 months | 16 | 12 | 23 | 21 |
| 1 year | 19 | 14 | 46 | 20 |
| 2 years | 13 | 14 | 13 | 19 |

*p*-values from *χ*^2^ or Fischer’s exact test between specific disease group versus HC are shown. AD: Alzheimer’s disease; bvFTD: behavioral variant frontotemporal dementia; DLB: dementia with Lewy bodies; HC: healthy control

**Supplemental Table 4.** Other sensory-motor symptoms experienced during the past 3 months, past year and past two years in different disease groups at different times before the diagnosis versus healthy controls at different times before filling in the questionnaire.

|  | AD  N = 64 | bvFTD  N = 38 | DLB  N = 17 | HC  N = 63 |
| --- | --- | --- | --- | --- |
| 10. Unexplained pain (not due to known conditions, such as arthritis) (%) | | | | |
| 3 months | 11 | 8 | 21 | 15 |
| 1 year | 9 | 6 | 13 | 18 |
| 2 years | 6 | 6 | 0 | 7 |
| 18. Feeling less interested in sex or more interested in sex (%) | | | | |
| 3 months | 19 | 41 | 39 | 26 |
| 1 year | 24 | 41 | 57 | 26 |
| 2 years | 10 ^(^*^p^* ^= 0.030)^ | 32 | 57 | 27 |
| 19. Finding it difficult to have sex when you try (%) | | | | |
| 3 months | 18 | 24 | 36 | 16 |
| 1 year | 15 | 29 | 62 ^(^*^p^* ^= 0.007)^ | 21 |
| 2 years | 6 | 23 | 57 ^(^*^p^* ^= 0.001)^ | 13 |
| 20. Feeling lightheaded, dizzy, or weak when standing up from sitting or lying (%) | | | | |
| 3 months | 20 | 11 | 42 ^(^*^p^* ^= 0.030)^ | 13 |
| 1 year | 15 | 19 | 21 | 17 |
| 2 years | 11 | 11 | 14 | 10 |
| 21. Falling (%) | | | | |
| 3 months | 5 | 9 ^(^*^p^* ^= 0.049)^ | 33 ^(^*^p^* ^< 0.001)^ | 0 |
| 1 year | 8 | 19 ^(^*^p^* ^= 0.039)^ | 43 ^(^*^p^* ^= 0.004)^ | 5 |
| 2 years | 4 | 24 ^(^*^p^* ^= 0.009)^ | 27 ^(p = 0.025)^ | 5 |
| 27. Swelling of your legs (%) | | | | |
| 3 months | 5 ^(p = 0.025)^ | 6 | 21 | 20 |
| 1 year | 8 ^(p = 0.020)^ | 6 | 21 | 19 |
| 2 years | 7 | 11 | 7 | 14 |
| 28. Excessive sweating (%) | | | | |
| 3 months | 7 ^(^*^p^* ^= 0.011)^ | 11 | 29 | 25 |
| 1 year | 8 ^(p = 0.020)^ | 11 | 14 | 25 |
| 2 years | 4 ^(^*^p^* ^= 0.004)^ | 11 | 0 | 23 |
| 29. Double vision (%) | | | | |
| 3 months | 2 | 6 | 25 ^(^*^p^* ^= 0.004)^ | 0 |
| 1 year | 7 ^(p = 0.049)^ | 3 | 14 ^(p = 0.035)^ | 0 |
| 2 years | 2 | 3 | 7 | 0 |

*p*-values from *χ*^2^ or Fischer’s exact test between specific disease group versus HC are shown.

AD: Alzheimer’s disease; bvFTD: behavioral variant frontotemporal dementia; DLB: dementia with Lewy bodies; HC: healthy control

**Non-Motor Symptom Questionnaire**

Chaudhuri KR, Martinez-Martin P. Quantitation of non-motor symptoms in Parkinson’s disease. *Eur J Neurol* 2008; 15: 2–8.
